# The Treatment of Disinfection of Scarlet Fever by Antiseptic Inunction

**Published:** 1893-06-24

**Authors:** J. Brendon Curgenven

**Affiliations:** Teddington Hall, S.W.


### Editor's Letter-Box.

[Our correspondents are reminded that prolixity is a great bar to publication, and that brevity of style and conciseness of statement greatly facilitate early insertion.]

### THE TREATMENT OF DISINFECTION OF SCARLET FEVER BY ANTISEPTIC INUNCTION.

Sir,—Observing an article in your journal by Dr. Bertram Rogers on inunction in scarlet fever, who, on the strength of one case, seeks to prove the failure of the method of treatment I have recommended, I desire to direct your attention to the utter inadequacy of such a mode of testing any treatment, especially the treatment of such an infectious disease as scarlet fever. His observations would have been of more value if he had treated a series of cases before communicating his experience to the profession. I first published my experience, obtained from the treatment of twenty-six cases, but the critics told me that was quite inadequate to establish its success. In this I acquiesced, and at the end of another year I gave the results of the treatment in a great number of cases furnished me by other medical men and heads of schools.

Dr. Rogers' case from the beginning was attended by certain elements leading to failure. He was not able to discover the original source of the infection, and possibly the servant nurse may have taken it from the same undiscovered source, or from some article of clothing that had escaped disinfection. Again, the hospital nurse was with the patient before the use of the oleusaban, and clearly took the infection soon after her arrival. It does not appear that he took any steps to disinfect those who had been in contact with the patient, and I cannot reconcile the two following passages in his communication. "No other member of the family took the disease," and "the only person unprotected by a previous attack of scarlet fever who escaped the infection was the medical man." I have always advised the disinfection of those who have been exposed to the infection by keeping them in an atmosphere charged with the vapour of the disinfectant for three days. All clothing, &c., should also be carefully disinfected by being sprinkled with the oleusaban, and shut up closely in a box or cupboard for a few days. The excretions should also be disinfected before removal from the room. I do advise the administration of the oleusaban eucalyptus or the globulus oil in three to six drop doses on sugar or in mucilage (*Medical Magazine*, page 739), and there is no necessity for the use of the biniodide of mercury nor sanitas. The oleusaban, which is a much more powerful antiseptic than the latter, can be applied to the throat by spray or brush. It consists of essential oils and camphors, and is not oily like olive oil. It is as clear as spirit, then how can it be an emulsion. A drop of water falls to the bottom of the bottle like a drop of mercury and remains there. The "B" oleusaban is an emulsion, and this I have used for closets, commodes, baths, &c., as it will mix with water. This should be mixed with the water used for rinsing or washing infected or soiled linen, for washing floors, furniture, utensils, &c.—essentials to be considered in the disinfection of the surroundings of a patient.—I am, dear sir, your obedient servant,

J. Brendon Curgenven.

Teddington Hall, S.W., June 8th, 1893.

